# Once-daily supplementation with pre-meal whey protein lowers breakfast postprandial glucose levels in women with GDM throughout the third trimester: a randomised, controlled, clinical trial

**DOI:** 10.1007/s00125-025-06587-0

**Published:** 2025-11-07

**Authors:** Stine Smedegaard, Nikolaj Rittig, Per G. Ovesen, Louise B. Suder, Janni H. Knudsen, Lise H. Brunsgaard, Ulla Kampmann

**Affiliations:** 1 Department of Clinical Medicine, Aarhus University, Aarhus, Denmark; 2 Steno Diabetes Center Aarhus, Aarhus University Hospital, Aarhus, Denmark; 3 Department of Endocrinology and Internal Medicine, Aarhus University Hospital, Aarhus, Denmark; 4 Department of Obstetrics and Gynecology, Aarhus University Hospital, Aarhus, Denmark; 5 Arla Foods Ingredients Group P/S, Viby, Aarhus, Denmark

**Keywords:** GDM, Gestational diabetes mellitus, Glycaemic management, Glycaemic variability, Postprandial glucose, Pregnancy, Protein supplementation, Randomised controlled trial, Whey protein

## Abstract

**Aims/hypothesis:**

This study aimed to investigate how pre-meal whey protein (WP) supplementation throughout the third trimester of pregnancy affects glycaemic and metabolic outcomes in women with gestational diabetes mellitus (GDM). The hypothesis was that WP, when administered as a pre-meal 30 min before breakfast daily, lowers glycaemic variability (primary outcome: CV%).

**Methods:**

In a double-blinded, randomised, placebo-controlled, parallel trial, 62 women with GDM were randomised to receive 20 g WP isolate/day or placebo 30 min before breakfast throughout the third trimester. Participants were randomly assigned (www.randomiser.org) to WP or placebo using a computer-generated list. Allocation was concealed with sealed strips. Participants, caregivers, investigators and outcome assessors were masked, except the dietitian providing dietary guidance. Eligibility criteria included GDM, normotension and age ≥18 years. Exclusion criteria included special dietary regimens ≥1 month, daily protein supplements, food allergies, glucose-metabolism-affecting drugs, twin pregnancies, polycystic ovary syndrome, severe comorbidity, hyperemesis or non-breakfast eaters. The study included laboratory visits, home-based measurements under controlled-living and free-living conditions during the early and late third trimester, and follow-up at delivery. Glucose levels were assessed using continuous glucose monitoring.

**Results:**

A total of 29 women were randomised to placebo and 33 were randomised to WP, with 25 in the placebo group and 30 women in the WP group completing the study. In the WP group, the 1 h postprandial glucose following breakfast was −20% (95% CI −28%, −11%) lower in the early and −15% (95% CI −24%, −5%) lower in the late third trimester compared with the placebo group under controlled conditions. Similarly, the 1 h postprandial glucose was −14% (95% CI −23%, −4%) lower in the early and −8% (95% CI −18%, 3%) lower in the late third trimester under free-living conditions. Glycaemic variability was lower in the WP group under controlled-living conditions. The mean amplitude of glycaemic excursions (MAGE) was lower during both the early and late third trimester, and the SD and CV% were lower during the early third trimester (all *p*<0.05). Time in range (proportion of time spent with glucose levels 3.5–7.8 mmol/l) was lower during free-living in the late third trimester (*p*=0.05).

**Conclusions/interpretation:**

Pre-meal WP improves glycaemic management and reduces glucose variability in women with GDM under controlled-living and free-living conditions. Future research should evaluate whether WP can delay or prevent pharmacological treatments such as insulin initiation.

**Trial registration:**

ClinicalTrials.gov NCT04767880

**Funding:**

Department of Clinical Medicine, Aarhus University and Arla Foods Ingredients Group P/S (Agr-2020–731–12107).

**Graphical Abstract:**

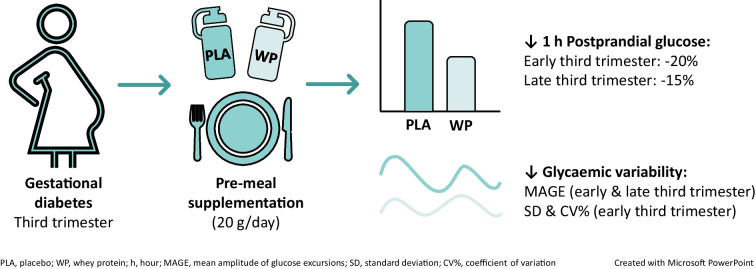

**Supplementary Information:**

The online version contains peer-reviewed but unedited supplementary material available at 10.1007/s00125-025-06587-0.


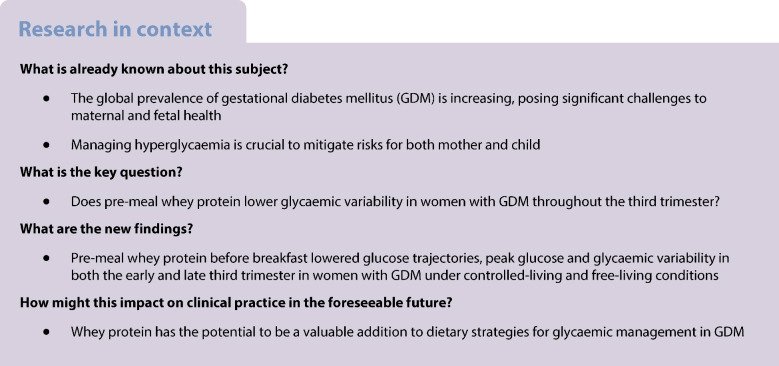


## Introduction

The prevalence of gestational diabetes mellitus (GDM) is increasing worldwide, posing a significant challenge to maternal and fetal health [1]. The IDF reported a global prevalence of hyperglycaemia in pregnancy of 19.7% in 2024, with 80% of the incidences being due to GDM [2]. However, these numbers vary depending on the population studied and the diagnostic criteria applied. GDM is associated with maternal hyperglycaemia that induces fetal hyperglycaemia, which in turn triggers fetal hyperinsulinaemia, leading to macrosomia. Macrosomia significantly increases the likelihood of adverse pregnancy outcomes, including prolonged labour, Caesarean delivery, shoulder dystocia and brachial plexus trauma [3, 4]. The implications of GDM extend beyond delivery, as women with GDM face a sevenfold increased risk of developing type 2 diabetes later in life compared with women with normal glucose tolerance (NGT) in pregnancy [5, 6]. Moreover, the metabolic consequences of GDM are intergenerational; children born to mothers with GDM face an increased risk of obesity, early-onset type 2 diabetes and the metabolic syndrome [7–9]. These risks underscore the urgency of effective treatment and management of GDM.

Dietary habits and sedentary lifestyles significantly contribute to the development of insulin resistance, a key factor in conditions such as the metabolic syndrome, obesity, type 2 diabetes and GDM. Current treatments for GDM primarily focus on improving dietary habits through professional nutritional guidance. However, despite these efforts, up to 50% of women with GDM require insulin therapy [4]. Although insulin is effective in lowering blood glucose levels, it poses several challenges, including psychological stress, high financial burden and an increased risk of hypoglycaemia and weight gain [10]. Given that insulin is the only approved pharmacological treatment for GDM in many countries, there is a growing need for non-pharmacological interventions to manage hyperglycaemia safely and effectively.

Pre-meals are smaller meals ingested before a main meal to lower postprandial blood glucose. Whey protein (WP) pre-meals lower postprandial blood glucose levels in healthy individuals, individuals with the metabolic syndrome and individuals with type 2 diabetes [11–19]. The mechanisms include increasing insulin and gut hormone levels as well as lowering gastric emptying [12, 20]. Additionally, WP has been associated with improved insulin sensitivity, prolonged satiety, reduced BP and favourable effects on lipid and bone metabolism [21, 22]. We recently showed that these acute metabolic effects on glucose levels also apply to women with GDM [23], and another study has demonstrated that casein, another milk protein, also has glucose-lowering effects following short-term pre-meal consumption [24]. Protein supplementation during pregnancy has been investigated in a limited number of clinical trials. However, these interventions have rarely lasted longer than 1 week [25–28]. This short duration, combined with the frequent lack of neonatal outcome data, significantly limits the clinical relevance of the findings. As a result, it remains unclear whether the glucose-lowering effect of milk proteins is sustained throughout pregnancy or whether this approach is both effective and safe as a treatment strategy for GDM. Given the clear lack of longer-term intervention studies in this population, the present study seeks to investigate the glycaemic effects of prolonged pre-meal WP consumption in women with GDM during the third trimester, focusing on glycaemic variability (primary outcome: CV%) along with potential effects on delivery and neonatal outcomes.

## Methods

### Study approval

The study was registered at ClinicalTrials.gov (registration no. NCT04767880), approved by the regional ethics committee (1-10-72-326-20), and complied with the Danish Data Protection Agency regulations. Women provided written informed consent.

### Participants

The study included women with GDM, defined by Danish criteria (glucose ≥9 mmol/l 2 h after a 75 g OGTT). They were normotensive and ≥18 years old. Exclusion criteria included special dietary regimens ≥1 month at inclusion, use of daily protein supplements, food allergies, glucose-metabolism-affecting drugs including insulin, non-Danish speakers, twin pregnancy, polycystic ovary syndrome, severe comorbidity, hyperemesis or non-breakfast eaters. Recruitment occurred after the women were diagnosed with GDM at the outpatient clinics of Aarhus University Hospital (AUH) and the regional hospitals in Horsens, Randers, Viborg and Gødstrup between December 2021 and August 2023.

### Design and protocol

The trial was a placebo-controlled, randomised, double-blinded, parallel study. Women were enrolled after diagnosis of GDM and started the trial between gestational week 28 + 0 and 32 + 6 and continued until delivery. The study consisted of two study days (one in the early third trimester and one in the late third trimester of pregnancy) performed at the Steno/Medical Aarhus Research Laboratory, AUH, Denmark, followed by four days of monitoring at home and data collection at birth. WP consumption started on the first day at home.

### Intervention, allocation and blinding

The primary investigator recruited and allocated participants to either placebo or WP using www.randomizer.org (version 4.0) [29]. The computer software generated a randomisation list from which participants were allocated to either WP or placebo. Allocation was concealed using sealed paper strips, which were removed only after a participant was enrolled and assigned a study ID. The intervention consisted of a WP isolate (WPI; Lacprodan ISO.WaterShake) and contained 20 g of protein, while the placebo compound was a no-energy drink with similar appearance and taste. The powder was produced and packed in small non-opaque sachets with three-digit codes. The pre-meals were mixed with 200 ml of tap water in a protein shaker. Participants consumed the pre-meals 30 min before breakfast every day until delivery. Pre-meal supplementation was administered in the morning, as the Nordic Nutrition Recommendations advise intervention at breakfast due to observed postprandial hyperglycaemia in the morning [30]. Additionally, supplementation was limited to once daily to ensure that protein intake did not exceed the recommended maximum of 35 energy % (E%) [31]. Participants, investigators, outcome assessors and trial staff (except the dietitian) were blinded to the interventions until all statistical analyses had been performed.

### Study days in the laboratory

Participants attended two study days in the laboratory: one at baseline during the early third trimester (between gestational week 28 + 0 and 32 + 6); and one during the late third trimester at gestational week 36. After fasting for 10–12 h, they underwent BP measurements and blood sampling, and were weighed and completed questionnaires on gastrointestinal symptoms. Before leaving, they received guidance from a dietitian to ensure equal macronutrient composition (especially protein) in the two groups.

### Study days at home

Participants were equipped with continuous glucose monitors and activity monitors for 4 days. During the first 2 days, participants were provided with standardised meals according to their energy requirements (9623, 10,873 or 12,128 kJ [2300, 2600 or 2900 kcal]), with identical protein content across groups (controlled conditions). Furthermore, they were asked to avoid strenuous exercise. During the next 2 days, participants were allowed to exercise freely and choose their own diet (free-living conditions). During all 4 days, they kept a diet diary and were instructed to avoid any food intake 3 h after their breakfast.

### At delivery

Cord blood was sampled immediately after delivery of the infant and placenta (at AUH) and maternal blood samples were taken within 3 days postpartum. Participants completed questionnaires on gastrointestinal symptoms within 1 week. Compliance was assessed through sachet counts, and participants were asked about their perception of group allocation. Women also reported their gestational weight gain (GWG).

### Study participant involvement

Meal plans were developed using patient surveys from another clinical study focusing on the acute effects of WP pre-meals in women with GDM [23]. The survey included food preferences such as breakfast habits, the occurrence of morning sickness, and preferred breakfast foods during pregnancy among 19 women with GDM. This approach aimed to enhance dietary compliance by incorporating foods that women with GDM prefer.

### Provision of standardised meals for controlled-living conditions

During the first 2 days at home (controlled-living conditions), all participants received the same standardised breakfast, consisting of 55 g oats, 200 ml 0.5% milk, 27 g raisins and 50 g banana. The remaining meals were adjusted based on individual energy requirements of 9623, 10,873 or 12,128 kJ (2300, 2600 or 2900 kcal) and group allocation (WP or placebo). The Institute of Medicine (IOM)’s Equations to Estimate Energy Requirements for pregnant women were used to calculate the energy need [31]. The meals were designed to fulfil a macronutrient profile of 55 E% carbohydrates (including dietary fibre), 25 E% fat, and 20 E% protein (including the WP), in alignment with nutritional guidelines set by the IOM and the Nordic Nutrition Recommendations [30, 31].

### Dietary guidance during free-living conditions

During the third and fourth day at home (free-living), the dietitian provided meal plans with various meal options based on the survey mentioned above. The women were provided with printed meal plans and received nutritional guidance three or four times during the study. They were guided to consume isonitrogenous diets (15–25 E% protein), ensuring that both groups received the same amount of protein regardless of intervention allocation. To facilitate this, the dietitian was not blinded to the interventions. The aim was for the women to eat 54 E% carbohydrates (acceptable: 47–57 E%), 20 E% protein (acceptable: 15–25 E%) and 26 E% fat (acceptable: 21–31 E%). This approach ensured that any effect on blood glucose was due to WP supplementation and not overall differences in total daily protein intake.

### Diet diaries

Participants registered their mealtimes and what they ate during the 4 days of monitoring at home (controlled-living and free-living conditions). Registration also occurred on two random days: one between the first and the second laboratory study days and one between the second laboratory study day and delivery. The overall energy content and macronutrient composition was calculated using www.Sundbid.dk, a Danish website for meal registration [32].

### Blood analysis

On the laboratory study days, blood was sampled in the preprandial state. At delivery, the prandial status was not controlled for. Plasma glucose was taken in fluoride tubes, immediately centrifuged, and then measured using a YSI 2300 model Stat Plus glucose analyser (YSI Incorporated, Yellow Springs, OH, USA). Serum insulin and C-peptide were analysed using the ELISA technique with commercial kits (Mercodia, Sweden). Samples were processed and stored for batch analysis at study completion. Cholesterol and HbA_1c_ were measured immediately at either the Department of Clinical Biochemistry, AUH (samples taken on laboratory study days) or local Departments of Clinical Biochemistry (samples taken at delivery).

### Continuous glucose monitoring

Glucose levels were measured continuously using a continuous glucose monitoring (CGM) system from Dexcom G6 (Dexcom, San Diego, CA, USA), provided by Healthlink Europe, the Netherlands. The monitoring device recorded glucose concentrations every 5 min via a sensor placed subcutaneously on the upper arm. Data were transmitted to a receiver for storage, with glucose levels concealed from participants to maintain blinding. After the study days, the raw data were extracted using the software CLARITY (v3.32.0, Dexcom).

### Activity monitoring

Physical activity and energy expenditure were measured using a combined accelerometer and heart rate (HR) monitor, Actiheart 5 (AH) from CamNtech, Cambridge, UK. Accelerometry data were sampled at 32 Hz, while HR was recorded as inter-beat intervals. Data analysis was performed using Actiheart software (version 5.1.10; CamNtech), which provided estimates on activity counts, activity energy expenditure (AEE), total energy expenditure (TEE), mean HR and maximum HR. The mean HR and maximum HR <50 beats/min, and activity counts/min <1 were treated as missing values, as these were obvious errors due to device failure, loose electrodes or units not being worn.

### Power calculation

We performed a power calculation with a significance level of 0.05, a power of 80%, an expected change in CV% (main outcome, a measure of glycaemic variability) of 5% points between groups, and an SD of 6.09. This difference is in line with the change seen in glycaemic variability when introducing diet treatment in GDM pregnancies [33] and the SD of glycaemic variability in non-insulin-dependent GDM pregnancies [34]. This resulted in *n*=50; however, to improve power and account for potential dropouts and missing data, a sample size of *n*=62 was planned.

### Statistics

Statistical analyses and figures were conducted using R (version 4.4.1; R Foundation for Statistical Computing, Vienna, Austria). Data are presented as medians with ranges or means with 95% CI. Statistical significance was defined as *p*<0.05.

Data measured once were analysed with an unpaired *t* test. In case of unequal variance, Welch’s test was applied. In case of non-normality, the Mann–Whitney *U* test was applied. Fisher’s exact test was used for categorical data.

Data with repeated measures were analysed using a mixed-effects model. Fixed effects included intervention, pregnancy period (early or late third trimester), setting (controlled-living or free-living), time when relevant and their interactions. Random effects included participant, participant nested within pregnancy period, participant nested within pregnancy period nested within setting, and site of recruitment (hospital). Model assumptions were validated by inspecting QQ plots for normality and plots of standardised vs fitted residuals for homogeneity of variance. A generalised linear model was applied to categorical data.

CGM data were processed for analysis using the R package ‘cgmanalysis’ [35]. Data gaps of <30 min were addressed using linear interpolation. The derived CGM parameters included mean glucose, maximum daily glucose, and measures glycaemic variability (SD, CV% and mean amplitude of glucose excursions [MAGE]). Time in range (TIR) was defined as the proportion of time that glucose levels were within the recommended target range of 3.5–7.8 mmol/l, as specified by clinical guidelines on treatment targets during pregnancy [36]. A 24 h day was defined as the period from 00:00 to 23:59 hours. Sensitivity analyses were performed excluding days with less than 80% data completeness. The trapezoidal rule was used to calculate the incremental AUC (iAUC) [37].

Data were analysed according to the intention-to-treat principle.

## Results

### Participants

**Fig. 1:**
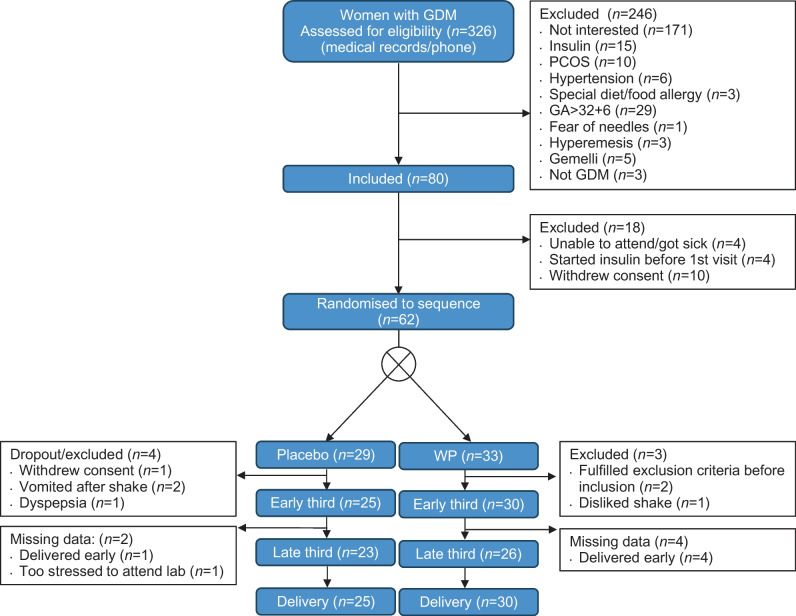
Flow chart of participant enrolment and randomisation. Early third refers to the early third trimester, and late third refers to the late third trimester. GA, gestational age; PCOS, polycystic ovary syndrome

Thirty-three participants were randomised to receive WP, and 29 to placebo. Three participants dropped out of the WP group, and four dropped out of the placebo group (Fig. 1). Baseline characteristics are presented in Table 1. The study sample represents women with GDM in the Central Region of Denmark; information on socioeconomic status was unavailable.

**Table 1:** Baseline characteristics

Characteristic	WP (*n*=30)	Placebo (*n*=25)	Median difference (95% CI)	*p* value^a^
Age, years	33 (24–38)	33 (22–40)	−1 (−2, 3)	0.720
Pre-pregnancy weight, kg	75 (48–130)	74 (50–103)	1 (−17, 4)	0.217
Mean pre-pregnancy weight, kg^b^	81.2 (73.1, 89.3)	75.7 (69.5, 81.9)	5.5 (−17, 4)	0.217
Pre-pregnancy BMI, kg/m^2^	28.7 (18.9–46.3)	26.6 (19.3–36.6)	2.1 (−5.8, 1.0)	0.162
Height, cm	165 (152–184)	165 (153–182)	−0.01 (−0.04, 0.04)	0.896
Parity, *n*	1 (0–3)	1 (0–2)	0 (−1, 1)	0.792
Ethnicity, *n*^c^			−	0.441
White	25	23		
Other	5	2		
Smoking, *n*				0.101
No	21	17		
Current	4	0		
Prior	5	8		
120 min glucose at diagnostic OGTT, mmol/l	10.0 (9.0–11.6)	9.8 (9.0–11.4)	0.2 (−0.8, 0.1)	0.114
GA at diagnosis, days	196 (122–210)	189 (73–210)	8 (−29, 38)	0.293

Data are presented as medians with ranges unless specified otherwise; differences are presented as median differences with 95% CI

^a^As all data were normally distributed, either in their raw form or after log transformation, a *t* test (comparing means) was used to assess differences between groups

^b^Mean with 95% CI

^c^Ethnicity was self-reported

GA, gestational age

### Metabolic profiling at the early and late third trimester, delivery and neonatal outcomes

The week of gestation, weight change during pregnancy, blood test results from the early (baseline) and late third trimester, delivery and neonatal outcomes are presented in Table 2.

**Table 2:** Results from early and late third trimester, delivery and neonatal outcomes

Outcome^a^	WP	Placebo	Mean difference (95% CI)	*p* value
Early third trimester				
No. of participants	30	25		
GA, days	216 (211, 221)	213 (208, 218)	3 (−2, 8)	0.500
Weight, kg	88.5 (81.1, 95.8)	82.8 (77.2, 88.5)	5.6 (−3.3, 14.6)	0.212
Fasting glucose, mmol/l	4.8 (4.6, 5.0)	4.8 (4.6, 5.0)	0 (−0.2, 0.3)	0.541
HbA_1c_, mmol/mol	33 (32, 34)	32 (31, 33)	1 (−1, 3)	0.204
HbA_1c_, %	5.2 (5.1, 5.3)	5.1 (4.9, 5.2)	0.1 (−0.2, 0.4)	0.457
Insulin, pmol/l	66.7 (53.4, 80.1)	59.5 (44.2, 74.9)	7.2 (−13.2, 28.0)	0.476
HOMA-IR	2.5 (1.9, 3.1)	2.2 (1.5, 2.8)	0.3 (−0.5, 1.2)	0.412
Late third trimester				
No. of participants	28	23		
GA, days	254 (253, 255)	255 (253, 256)	−1 (−6, 5)	0.829
Δ GWG, kg	2.2 (1.0, 3.4)	2.0 (0.6, 3.6)	0.16 (−1.98, 1.66)	0.859
Δ Total GWG, kg	8.8 (6.6, 10.9)	9.2 (6.7, 11.7)	−0.41 (−2.77, 3.6)	0.795
Δ Fasting glucose, mmol/l	−0.1 (−0.3, 0.1)	−0.2 (−0.3, 0.01)	0 (−0.2, 0.3)	0.756
Δ HbA_1c_, mmol/mol	1.3 (0.8, 1.9)	1.8 (1.0, 2.6)	−0.1 (−1.1, 0.9)	0.831
Δ HbA_1c_, %	0.0 (−0.2, 0.2)	0.2 (0.1, 0.2)	−0.2 (−0.4, 0.2)	0.833
Δ Insulin, pmol/l	−1.1 (−10.9, 8.8)	−0.001 (−12.6, 12.6)	−1.1 (−16.6, 14.5)	0.892
Δ HOMA-IR	−0.1 (−0.5, 0.4)	−0.1 (−0.6, 0.4)	0.02 (−0.6, 0.7)	0.959
Delivery				
No. of participants	30	25		
GA delivery, days	273 (269, 277)	278 (274, 281)	−5 (−10, 1)	0.084
Δ Total GWG, kg	9.0 (6.9, 11.2)	9.8 (7.4, 12.3)	−0.8 (−4.0, 2.4)	0.606
Δ HbA_1c_, mmol/mol	2.7 (1.9, 3.4)	2.7 (1.8, 3.5)	−0.4 (−1.4, 0.6)	0.411
Δ HbA_1c_, %	0.1 (−0.1, 0.4)	0.1 (−0.2, 0.4)	0 (−0.3, 0.3)	0.369
No. of days with treatment	57 (51, 62)	65 (60, 69)	−8 (−15, −1)	0.033
Compliance, %	95 (68, 100)	94 (80, 107)	1 (−5, 7)	0.681
Newborn				
No. of newborns	30	25		
Birthweight, g	3460.9 (3285.3, 3636.5)	3610.5 (3425.9, 3795.2)	−150 (−399, 100)	0.235
Abdominal circumference, cm	33.3 (32.4, 34.1)	33.4 (32.4, 34.4)	−0.1 (−1, 1)	0.833
Length, cm	50.6 (49.5, 51.7)	52.0 (50.9, 53.0)	−1.4 (−3, 0.1)	0.071
*z* score birthweight	0.19 (−0.15, 0.54)	0.20 (−0.18, 0.57)	−0.005 (−0.50, 0.51)	0.983
Cord blood				
No. of samples	10	9		
Insulin, pmol/l	140 (19, 260)	53 (13, 93)	87 (−37, 209)	0.151
C-peptide, pmol/l	686 (314, 1058)	366 (233, 498)	320 (−62, 703)	0.093

Data are presented as means with 95% CI

The participants attended the laboratory in the preprandial state in the early third trimester (~30 weeks of gestation) and again in the late third trimester (36 weeks of gestation). During delivery or a maximum of 3 days after, women had the final follow-up. Data on neonatal outcomes were collected at delivery

^a^Δ represents changes from baseline (early third trimester) to the specified time point (late third trimester or delivery); Δ Total indicates the change from pre-pregnancy to the specified time point (late third trimester or delivery)

GA, gestational age

GWG and HOMA-IR scores were similar between groups. Compliance exceeded 94% in both groups. The placebo group underwent treatment for 8 days longer than the WP group (*p*=0.033); the mean treatment time was 8 weeks (95% CI 5 weeks, 12 weeks) for the WP group and 9 weeks (95% CI 6 weeks, 13 weeks) for the placebo group. Newborn birthweight and *z* score did not differ between groups. Cord blood C-peptide levels were higher in the WP group than in the placebo group (*p*=0.093), while insulin levels were comparable between groups (*p*=0.151).

### CGM

Sensor data were available for 98.8% (range 57–100%) of the expected monitoring period. The remaining gaps were due to factors such as the receiver being out of range or sensor failure. After imputation by linear interpolation, 99.3% (range 57–100%) of sensor data were available for analysis. One participant in the WP group had 1 day with <70% sensor data available and one in the placebo group had 1 day with <80% sensor data availability. All other days with CGM recordings had ≥84% sensor data availability.

**Fig. 2:**
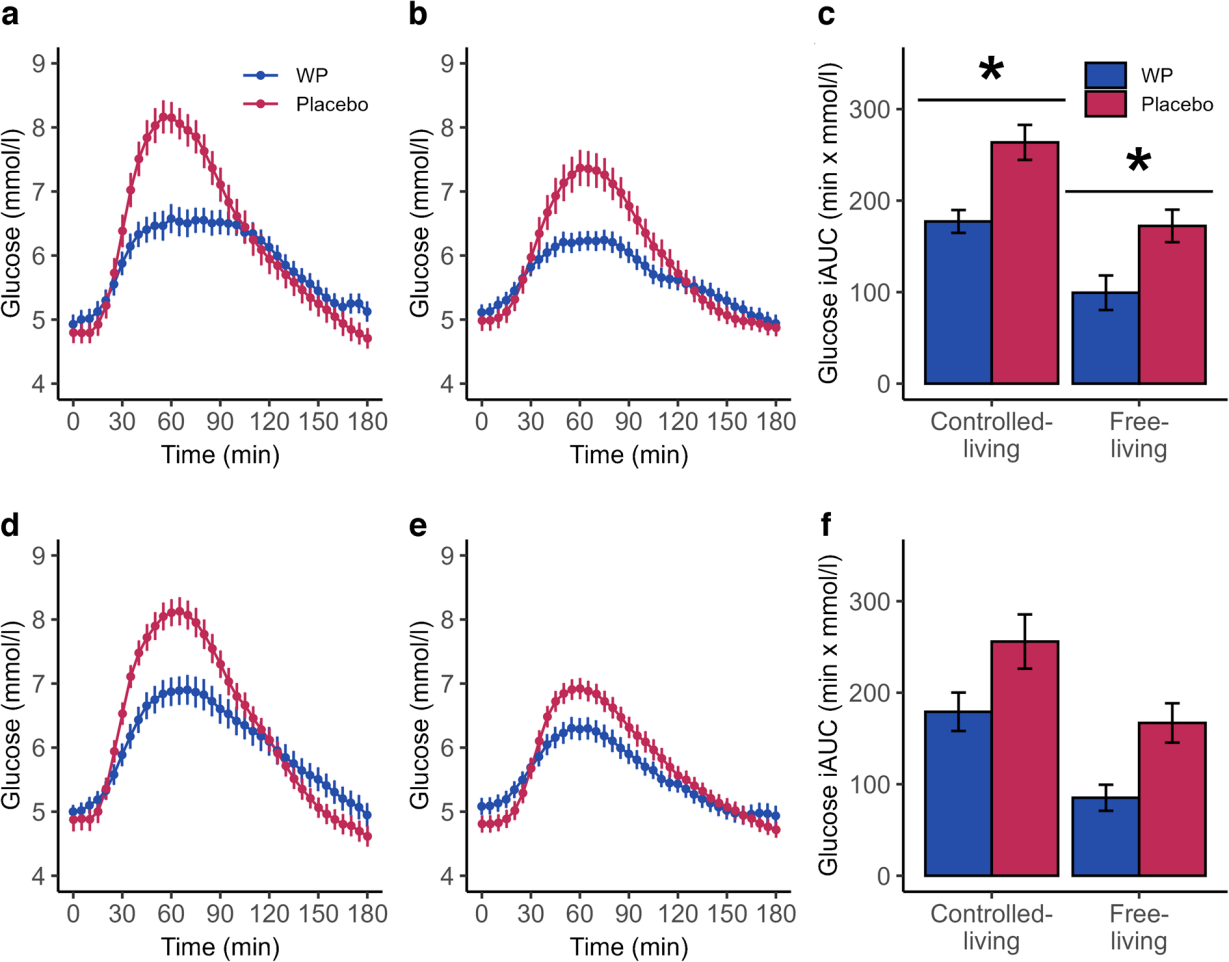
Glucose trajectories and iAUC. Women were randomised to consume either a no-energy placebo drink or 20 g WP 30 min before breakfast. In the early third trimester, participants were equipped with CGM and provided with pre-meals and standardised meals for 2 days under controlled-living conditions: glucose trajectories (**a**) and iAUC (**c**) are shown. This was followed by 2 days of free-living, where participants were allowed to exercise and eat freely: glucose trajectories (**b**) and iAUC (**c**) are shown. Investigations were repeated in the late third trimester: glucose trajectories under controlled-living (**d**) and free-living conditions (**e**) and iAUC (**f**) are shown. Data are presented as means with SEs. Blue indicates pre-meal WP, and red indicates placebo. A mixed-effects model was used to evaluate the interaction between time and intervention (*p* value for time × intervention <0.001 in **a**, **b**, **d**, **e**), and pairwise comparisons were computed to assess differences in iAUC between placebo and WP pre-meals. *p<0.05

**Fig. 3:**
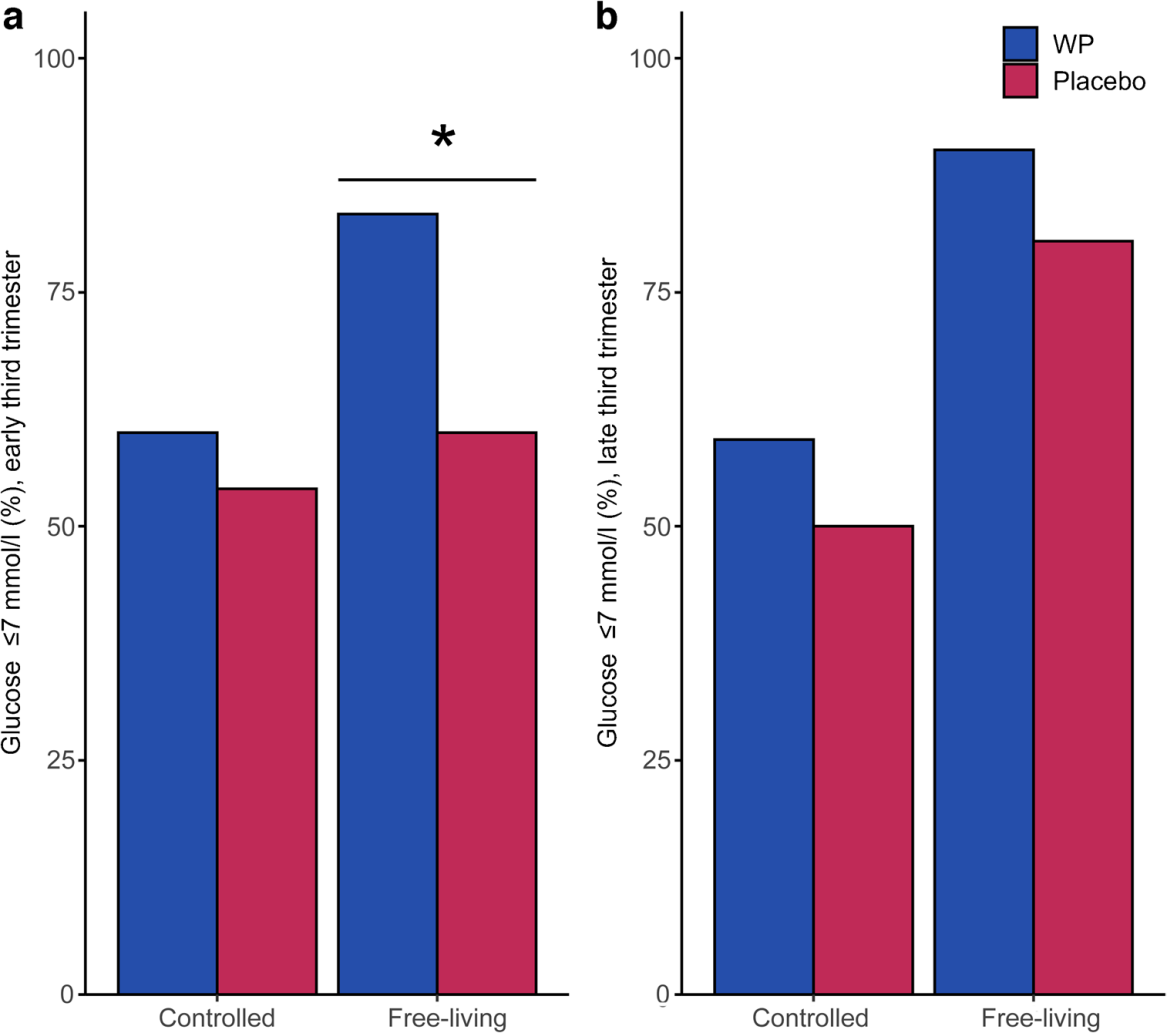
Proportion of women achieving treatment targets of ≤7 mmol/l glucose 90 min post-meal during the early (**a**) and late (**b**) third trimester. Data are presented as proportions in %. Blue indicates pre-meal WP, and red indicates placebo. A mixed-effects model was used to analyse data followed by pairwise comparisons for differences between placebo and WP. *p<0.05

Glucose trajectories showed a slower increase and a lower maximum after pre-meal WP ingestion under both controlled-living and free-living conditions during both early and late third trimester (Fig. 2a, b, d, e). In the WP group, the 1 h postprandial glucose following breakfast was −20% (95% CI −28%, −11%) lower in the early and −15% (95% CI −24%, −5%) lower in the late third trimester compared with the placebo group during the first 2 days with home monitoring under controlled-living conditions (Fig. 2a, d). Similarly, the 1 h postprandial glucose was −14% (95% CI −23%, −4%) lower in the early and −8% (95% CI −18%, 3%) lower in the late third trimester during the next 2 days of home monitoring under free-living conditions (Fig. 2b, e). The iAUC for glucose was lower in the WP group during both gestation periods but the difference was only statistically significant during the early third trimester (Fig. 2c, f). The proportion of women with glucose measurements ≤7 mmol/l at 90 min postprandially (initiation criteria for insulin treatment [38]) was significantly higher during free-living in the early third trimester in the WP group compared with placebo (50/60 measurements [83%] vs 30/50 [60%], *p*=0.019) (Fig. 3). Consistently, when considering women who maintained all their postprandial glucose values below this threshold, a larger proportion was observed in the WP group compared with placebo (22/30 women [73%] vs 11/25 [44%], *p*=0.03). Exact glucose levels at each time point are presented in electronic supplementary material (ESM) Table 1.

The mean glucose concentration was similar between groups, period of trimester (early and late third trimester), and setting (controlled- and free-living conditions) (Table 3). Glucose variability, assessed by SD and CV%, was lower in the WP group than in the placebo group during controlled-living conditions in the early third trimester (*p*=0.027 and *p*=0.046, respectively) (Table 3), and MAGE was lower during the first 2 days of home monitoring (controlled conditions) in both early and late third trimester in the WP group compared with the placebo group (*p*=0.003 and *p*=0.023, respectively) (Table 3). The TIR, time above range (TAR), and the time below range (TBR) were similar between groups under both controlled- and free-living conditions in both early and late third trimester (Table 3). After excluding days with <80% sensor data availability, TIR was lower in the WP group compared with the placebo group during free-living in the late third trimester (*p*=0.035).

**Table 3:** Effect of pre-meal WP on glycaemic management during the early and late third trimester

Variable	WP	Placebo	Mean difference (95% CI)	*p* value
Early third trimester, controlled living				
No. of participants	30	25		
TAR, % [hours:minutes]	3 (–1, 7) [0:43]	6 (2, 10) [1:26]	–3 (–7, 1) [−0:43]	0.147
TIR, % [hours:minutes]	85 (78, 91) [20:24]	83 (76, 89) [19:55]	2 (–5, 8) [0:29]	0.560
TBR, % [hours:minutes]	6 (3, 10) [1:26]	6 (2, 10) [1:26]	0 (–4, 4) [0:00]	0.984
Average glucose, mmol/l	5.3 (5.0, 5.7)	5.5 (5.1, 5.9)	–0.1 (–0.6, 0.3)	0.534
CV, %	18 (16, 19)	19 (17, 21)	–2 (–4, −0.03)	0.046
SD, mmol/l	0.9 (0.8, 1.0)	1.1 (0.9, 1.2)	–0.1 (–0.3, −0.02)	0.027
MAGE, mmol/l	1.9 (1.6, 2.2)	2.3 (2.0, 2.7)	–0.5 (–0.8, –0.2)	0.003
Sensor data availability, % [range]	99 (99, 100) [90–100]	99 (99, 100) [89–100]	0 (−4, 4)	0.994
Early third trimester, free-living				
No. of participants	30	25		
TAR, % [hours:minutes]	3 (–1, 7) [0:43]	7 (3, 11) [1:41]	–4 (–8, 1) [–0:58]	0.118
TIR, % [hours:minutes]	89 (83, 96) [21:22]	85 (78, 91) [20:24]	4 (–2, 11) [0:58]	0.181
TBR, % [hours:minutes]	2 (–1, 5) [0:29]	2 (–1, 6) [0:29]	0 (–5, 4) [0:00]	0.894
Average glucose, mmol/l	5.5 (5.1, 5.9)	5.6 (5.3, 6.0)	–0.2 (–0.6, 0.3)	0.496
CV, %	16 (14, 18)	17 (15, 19)	–1 (–3, 1)	0.398
SD, mmol/l	0.9 (0.8, 1.0)	0.9 (0.8, 1.1)	–0.1 (–0.2, 0.1)	0.320
MAGE, mmol/l	1.9 (1.5, 2.2)	2.0 (1.7, 2.4)	–0.2 (–0.5, 0.1)	0.254
Sensor data availability, % [range]	100 (97, 100) [59–100]	99 (98, 100) [84–100]	1 (−2, 5)	0.350
Late third trimester, controlled living				
No. of participants	28	23		
TAR, % [hours:minutes]	5 (1, 9) [1:12]	4 (0, 8) [0:58]	1 (–4, 5) [0:14]	0.723
TIR, % [hours:minutes]	83 (76, 89) [19:55]	87 (81, 94) [20:52]	–4 (–12, 2) [–0:57]	0.165
TBR, % [hours:minutes]	6 (3, 10) [1:26]	4 (0, 8) [0:58]	2 (–2, 7) [0:28]	0.264
Average glucose, mmol/l	5.4 (5.0, 5.7)	5.4 (5.0, 5.8)	–0.1 (–0.5, 0.4)	0.823
CV, %	19 (17, 21)	20 (18, 22)	–2 (–4, 0)	0.129
SD, mmol/l	1.0 (0.9, 1.1)	1.1 (0.9, 1.2)	–0.1 (–0.2, 0.05)	0.206
MAGE, mmol/l	2.0 (1.7, 2.3)	2.4 (2.0, 2.7)	–0.4 (–0.7, –0.1)	0.023
Sensor data availability, % [range]	99 (98, 100) [84–100]	99 (99, 100) [92–100]	0 (−2, 6)	0.373
Late third trimester, free-living				
No. of participants	28	23		
TAR, % [hours:minutes]	6 (2, 10) [1:26]	2 (–2, 6) [0:29]	4 (–0, 8) [0:57]	0.075
TIR, % [hours:minutes]	85 (79, 91) [20:24]	92 (85, 98) [22:05]	–7 (–14, 0) [–1:41]	0.050^a^
TBR, % [hours:minutes]	3 (–1, 6) [0:43]	1 (–3, 5) [0:14]	2 (–3, 6) [0:29]	0.510
Average glucose, mmol/l	5.6 (5.2, 6.0)	5.5 (5.1, 5.9)	0.1 (–0.3, 0.6)	0.554
CV, %	17 (15, 19)	17 (15, 19)	0 (–2, 2)	0.938
SD, mmol/l	0.9 (0.8, 1.1)	0.9 (0.8, 1.0)	0.0 (–0.1, 0.1)	0.804
MAGE, mmol/l	2.0 (1.6, 2.3)	2.0 (1.6, 2.3)	0.0 (–0.3, 0.3)	0.945
Sensor data availability, % [range]	100 (99, 100) [94–100]	100 (98, 100) [73–100]	0 (−4, 3)	0.779

Data are presented as means with 95% CI

Participants were investigated with CGM during the early third trimester (~30 weeks of gestation) and again in the late third trimester (36 weeks of gestation) under both controlled-living and free-living conditions

^a^*p*<0.05 after exclusion of days with less than 80% sensor data availability

### Activity

There were no differences in activity scores between the two groups (ESM Table 2). The participants had a physical activity level (PAL) between 1.45 and 1.54, which is characterised as ‘low active’ [31].

### Diet diary

Overall, participants reported comparable diets throughout the study (ESM Table 3). Women in the WP group reported consuming more protein and less fat compared with the placebo group (protein, WP 23% vs placebo 19%, *p*<0.001; fat, WP 32% vs placebo 36%, *p*=0.016) in the early third trimester. Furthermore, women in the WP group reported a higher intake of carbohydrates at breakfast compared with the placebo group (WP 51%, placebo 44%, *p*=0.049) during the late third trimester. On random days with food registration, the WP group reported eating less protein for breakfast (WP 20% vs placebo 23%, *p*=0.034), and more protein and less fat in their complete daily diets (protein, WP 23% vs placebo 20%, *p*=0.001; fat, WP 31% vs placebo 36%, *p*=0.002) compared with the placebo group.

### Blinding

Among the women who received WP, the majority (61%) were uncertain about their allocation, while 21% believed they received a placebo and 18% believed they had WP. Among the women who received the placebo, 50% were uncertain about their allocation; 46% believed they received a placebo and 4% thought they had WP.

### Side effects and obstetrical outcomes

There were no differences in gastrointestinal side effects or obstetrical outcomes (ESM Fig. 1, ESM Table 4).

## Discussion

This randomised clinical trial demonstrated that a 20 g WP pre-meal significantly lowered postprandial glucose trajectories compared with a no-energy placebo drink during the third trimester of pregnancy in women with GDM. The WP group more frequently achieved the treatment goal of glucose levels below 7 mmol/l at 90 min following a meal.

This study has notable strengths and limitations. Among its strengths, the double-blinded design ensured that participants were unaware of their group assignment. Additionally, all investigators remained blinded until all data analysis had been completed to avoid bias. The study assessed the effect of WP pre-meals twice during the third trimester, providing valuable insights into the effects of prolonged WP supplementation. Additionally, the study incorporated both controlled-living and free-living conditions, enhancing the generalisability of the findings. The significant effect of WP on glucose metabolism during free-living conditions, combined with a 95% compliance rate in the WP group, highlights its potential applicability in a real-world setting. A major strength of this study is the incorporation of CGM, which enables the collection of high-resolution glucose data at 5 min intervals while minimising the need for frequent capillary blood sampling. Nonetheless, the use of CGM in the context of GDM may not fully align with standard clinical practice, which predominantly relies on capillary glucose measurements. Moreover, the inherent delay in glucose equilibration between interstitial fluid and capillary blood should be accounted for when assessing whether postprandial glucose levels meet therapeutic targets.

The greatest effect of WP on glucose levels was evident during the early third trimester under both controlled-living and free-living conditions. The impact of pre-meal WP was less pronounced under free-living conditions in the late third trimester. Differences in dietary macronutrient composition and total energy content, as well as physical activity, were small and without statistical significance, therefore did not explain the observed difference in glucose levels. Data were analysed using the ‘intention-to-treat’ approach, whereby dropouts and missing data due to participants delivering before completing the second study phase may have contributed to the attenuated effects observed in the late third trimester. Furthermore, the natural progression of insulin resistance during pregnancy may have also influenced the findings [39–41].

Few studies have explored the impact of dietary protein supplementation during pregnancy for durations exceeding 1 week [24–28]. Two of these studies reported reduced fasting blood glucose [25, 26], while four demonstrated lower postprandial blood glucose levels [24, 27, 28]. This study observed no significant effect on fasting blood glucose; however, consistent with most prior studies, WP reduced postprandial glucose levels. Of particular interest, Li et al applied a mixed preload containing 7.6 g protein thrice daily, Feng et al administered 20 g of WP pre-meal, and Saleh et al used 8.5 g casein pre-meal twice daily [24, 26, 28]. Both Li et al and Feng et al reported reductions in 2 h postprandial blood glucose levels, while Saleh et al observed lower glucose concentrations within the first hour after the meal. The findings of our study align with these results, as the most pronounced effects were observed within the first hour after the meal. Additionally, a larger proportion of women in the WP group achieved glucose treatment targets of <7 mmol/l 90 min postprandially compared with the placebo group, especially during free-living conditions in the early third trimester. These findings suggest that pre-meal WP may serve as a potential non-pharmaceutical approach in managing hyperglycaemia in GDM and delaying or avoiding insulin therapy. However, no differences were observed in HbA_1c_, fasting glucose or HOMA-IR. This may reflect the single administration of the pre-meal and the regular contact with clinical dietitians, which likely helped participants achieve treatment targets regardless of intervention, reducing the potential for WP to exert additional effects.

CGM measures for glucose variability, including SD and CV%, were lower in the WP group but the difference was only statistically significant during the early third trimester under controlled-living conditions. MAGE was consistently lower in the WP group under both controlled-living and free-living conditions, with a statistically significant difference during controlled-living conditions compared with the placebo group. A study from 2014 evaluated the correlation between glycaemic variability and adverse pregnancy and neonatal outcomes and showed that increased MAGE was associated with higher birthweight and increased risk of preeclampsia as well as neonatal complications such as neonatal hypoglycaemia and being large for gestational age (LGA) [42]. On the contrary, two other studies found no significant correlation between glycaemic variability and LGA [43] or between glycaemic variability and birthweight or adverse pregnancy outcomes [34]. The impact of glycaemic variability on pregnancy outcomes is uncertain and awaits the outcome of future clinical trials and the broad use of CGM during pregnancy [34, 42, 43]. There were no differences in TIR (glucose 3.5–7.8 mmol/l), TAR or TBR between the groups. However, after excluding days with <80% sensor data availability, the WP group demonstrated a 7% lower TIR in the late third trimester under free-living conditions. There are no official recommendations regarding TIR in women with GDM; however, pregnant women with type 1 diabetes are advised to exceed >70% TIR and benefit from even modest improvements with just a 5% increase in TIR associated with better outcomes [44]. It is unknown whether these findings also apply to GDM; however, the observed 7% difference may be clinically meaningful and warrants further investigation.

WP is rich in leucine, an amino acid with insulinotropic properties [45, 46], which may cross the placenta and contribute to fetal hyperinsulinaemia and excessive fetal growth [47, 48]. While cord blood insulin and C-peptide levels were higher (but not statistically significantly different), no significant effect on infant weights, abdominal circumferences and *z* scores was found when comparing groups. Therefore, supplementation with 20 g WP seems to be safe during the third trimester of pregnancy.

Dietary intake was randomly recorded during the third trimester to assess adherence to dietary guidance and determine whether the effects were due to pre-meal WP consumption or adaptations in the diet (e.g. higher protein intake). A professional dietitian ensured that both groups maintained similar protein and energy intake, minimising dietary discrepancies. Despite this, minor differences in macronutrient composition were observed, though they were unlikely to have any clinical significance. The involvement of a dietitian represents both a strength and a limitation. While dietary guidance ensured standardised practices in compliance with guidelines in both groups, it may have attenuated the impact of WP.

In conclusion, pre-meal WP may improve glucose trajectories and glycaemic variability measured as MAGE in women with GDM during the third trimester of pregnancy. Future research is needed to evaluate the safety of WP and determine whether WP supplements can delay or prevent the initiation of pharmacological treatments such as insulin.

## Supplementary Information

Below is the link to the electronic supplementary material.

ESM (PDF 385 KB)

## Data Availability

Data are available on request from the corresponding author.

## Abbreviations

AUH: Aarhus University Hospital
CGM: Continuous glucose monitoring
GDM: Gestational diabetes mellitus
GWG: Gestational weight gain
HR: Heart rate
iAUC: Incremental AUC
IOM: Institute Of Medicine
LGA: Large for gestational age
MAGE: Mean amplitude of glucose excursions
TAR: Time above range
TBR: Time below range
TIR: Time in range
WP: Whey protein
