# Supplementary material for: Once-daily supplementation with pre-meal whey protein lowers breakfast postprandial glucose levels in women with GDM throughout the third trimester: a randomised, controlled, clinical trial
Source: Diabetologia. 2025 Nov 7;69(2):350–63. doi: 10.1007/s00125-025-06587-0 (PMC12779656; doi:10.1007/s00125-025-06587-0)
Supplement: Supplementary file 1 — ESM (PDF 385 KB) [file 125_2025_6587_MOESM1_ESM.pdf]

Electronic supplementary material (ESM)

Pre-meal	Period	Setting	0 min	60 min	90 min	120 min	180 min	Mean	Peak
WP	Early	Controlled	4.9 ± 0.2	6.6 ± 0.2*	6.5 ± 0.2	6.2 ± 0.2	5.1 ± 0.2	5.9 ± 0.2	7.8 ± 0.3*
Placebo	Early	Controlled	4.8 ± 0.2	8.2 ± 0.2	7.1 ± 0.3	5.9 ± 0.2	4.7 ± 0.2	6.2 ± 0.2	9.0 ± 0.4
WP	Early	Free	5.1 ± 0.1	6.2 ± 0.2*	6.1 ± 0.2	5.6 ± 0.2	4.9 ± 0.1	5.7 ± 0.1	7.4 ± 0.2*
Placebo	Early	Free	5.0 ± 0.2	7.4 ± 0.3	6.8 ± 0.2	5.7 ± 0.2	4.9 ± 0.1	5.9 ± 0.2	8.3 ± 0.4
WP	Late	Controlled	5.0 ± 0.1	6.9 ± 0.2*	6.6 ± 0.2	6.2 ± 0.2	5.0 ± 0.2	6.0 ± 0.2	7.9 ± 0.3*
Placebo	Late	Controlled	4.9 ± 0.2	8.1 ± 0.2	7.3 ± 0.2	6.1 ± 0.2	4.6 ± 0.2	6.3 ± 0.1	9.2 ± 0.3
WP	Late	Free	5.1 ± 0.1	6.3 ± 0.2	5.9 ± 0.2	5.4 ± 0.1	4.9 ± 0.2	5.5 ± 0.1	7.3 ± 0.3
Placebo	Late	Free	4.8 ± 0.1	6.9 ± 0.2	6.3 ± 0.2	5.6 ± 0.2	4.7 ± 0.1	5.7 ± 0.1	8.0 ± 0.2

ESM Table 1. Glucose after breakfast during controlled and free-living conditions. Data are presented as mean ± SE in mmol/L. Early refers to “early third trimester”, and Late refers to “late third trimester”. A mixed-effects model was used to analyse the data with pairwise comparisons for differences between the placebo and WP pre-meals at any given timepoint with * indicating $p < 0.05$.

Activity measures								
**Pre-meal**	**Period**	**Setting**	**AEE (kcal)**	**TEE (kcal)**	**PAL**	**Mean HR (BPM)**	**Max HR (BPM)**	**Activity (counts/min)**
WP	Early	Controlled	478 [392; 590]	2392 [2186; 2618]	1.46 [1.39; 1.55]	81 [77; 85]	104 [97; 112]	18 [15; 22]
Placebo	Early	Controlled	450 [358; 561]	2298 [2101; 2540]	1.45 [1.38; 1.54]	81 [77; 86]	107 [98; 116]	18 [15; 22]
WP	Early	Free	518 [420; 639]	2441 [2231; 2670]	1.51 [1.43; 1.6]	81 [77; 86]	107 [99; 116]	20 [17; 24]
Placebo	Early	Free	545 [433; 679]	2416 [2186; 2644]	1.52 [1.43; 1.6]	81 [77; 86]	107 [98; 116]	22 [18; 27]
WP	Late	Controlled	488 [395; 608]	2416 [2208; 2644]	1.46 [1.39; 1.55]	78 [74; 82]	105 [98; 113]	18 [15; 22]
Placebo	Late	Controlled	545 [433; 692]	2490 [2253; 2724]	1.54 [1.45; 1.63]	81 [77; 86]	109 [100; 118]	18 [15; 22]
WP	Late	Free	455 [365; 567]	2392 [2186; 2618]	1.46 [1.38; 1.54]	78 [74; 83]	103 [95; 111]	16 [13; 19]
Placebo	Late	Free	503 [395; 633]	2416 [2186; 2670]	1.51 [1.42; 1.6]	81 [77; 86]	109 [100; 118]	18 [15; 22]
**Difference in activity between WP and placebo**								
**Difference**	**Period**	**Setting**	**AEE (%)**	**TTE (%)**	**PAL (%)**	**Mean HR (%)**	**Max HR (%)**	**Activity (%)**
WP-placebo	Early	Controlled	7 [-20; 43]	4 [-6; 15]	1 [-6; 8]	-1 [-8; 6]	-3 [-11; 7]	-1 [-23; 27]
WP-placebo	Early	Free	-5 [-29; 27]	1 [-9; 13]	-1 [-8; 7]	-1 [-8; 6]	0 [-9; 11]	-9 [-29; 17]
WP-placebo	Late	Controlled	-10 [-34; 21]	-3 [-12; 8]	-5 [-11; 3]	-4 [-10; 4]	-3 [-12; 7]	-1 [-23; 28]
WP-placebo	Late	Free	-10 [-33; 23]	-1 [-11; 9]	-3 [-10; 4]	-4 [-10; 3]	-6 [-15; 4]	-10 [-31; 16]

ESM Table 2. Activity data from controlled and free-living conditions during the early and late third trimester. Data are presented as means with 95%CI; differences are presented as % median difference with 95%CI. The upper part of the table presents the activity measures for both groups of women (placebo and WP) at each pregnancy period (early and late third trimester) during the study days at home under controlled- and free-living conditions. The lower part presents the difference in activity measures between the placebo and WP group. Early refers to “early third trimester”, and Late refers to “late third trimester”. A mixed-effects model was used to analyse data, followed by pairwise comparisons for differences between placebo and WP. AEE, activity energy expenditure; TEE, total energy expenditure; PAL, physical activity level; HR, heart rate.

**Early third trimester**	**WP, *n*=30**	**Placebo, *n*=25**	**Mean difference**	***p***
Morning (kcal)	353 [321; 385]	354 [322; 385]	-1 [-55; 54]	0.986
Protein (E%)	19 [18; 21]	20 [19; 22]	-1 [-4; 2]	0.470
Carbohydrate (E%)	52 [49; 56]	48 [44; 52]	4 [-2; 11]	0.215
Fat (E%)	24 [21; 28]	28 [24; 32]	-4 [-10; 4]	0.361
Dietary fiber (E%)	4 [4; 4]	4 [3; 4]	0 [0; 1]	0.388
Full day (kcal)	1893 [1776; 2009]	1942 [1827; 2058]	-49 [-239; 131]	0.566
Protein (E%)	23 [22; 24]	19 [18; 20]	4 [2; 5]	**< 0.001**
Carbohydrate (E%)	42 [40; 43]	41 [39; 44]	1 [-3; 4]	0.743
Fat (E%)	32 [30; 34]	36 [33; 38]	-4 [-7; -1]	**0.016**
Dietary fiber (E%)	3 [3; 4]	4 [2; 6]	-1 [-2; 0]	0.124
**Late third trimester**	**WP, *n*=28**	**Placebo, *n*=23**	**Mean difference**	***p***
Morning (kcal)	324 [284; 363]	347 [313; 382]	-29 [-86; 29]	0.322
Protein (E%)	20 [18; 22]	22 [20; 24]	-2 [-5; 1]	0.255
Carbohydrate (E%)	51 [47; 55]	44 [40; 48]	7 [0; 14]	**0.049**
Fat (E%)	24 [20; 28]	30 [26; 35]	-6 [-11; 3]	0.084
Dietary Fiber (E%)	4 [4; 5]	3 [3; 4]	1 [0.06; 1]	**0.034**
Full day (kcal)	1899 [1781; 2016]	1909 [1791; 2026]	-10 [-226; 161]	0.740
Protein (E%)	22 [21; 23]	21 [20; 22]	1 [-0.4; 3]	0.129
Carbohydrate (E%)	42 [40; 44]	41 [39; 43]	-1 [-3; 4]	0.786
Fat (E%)	32 [30; 34]	34 [32; 36]	-2 [-5; 2]	0.295
Dietary fiber (E%)	3 [3; 4]	3 [3; 4]	0 [-1; 1]	0.934
**Random day**	**WP, *n*=30**	**Placebo, *n*=24**	**Mean difference**	***p***
Morning (kcal)	305 [268; 343]	323 [285; 361]	-18 [-83; 33]	0.393
Protein (E%)	20 [17; 22]	23 [20; 27]	-3 [-7; -0.3]	**0.034**
Carbohydrate (E%)	51 [47; 55]	45 [41; 49]	6 [-0.1; 13]	0.055
Fat (E%)	24 [20; 29]	28 [23; 33]	-4 [-13; 1]	0.294
Dietary Fiber (E%)	4 [3; 4]	4 [3; 4]	0 [0; 1]	0.527
Full day (kcal)	1838 [1723; 1954]	1782 [1665; 1899]	56 [-170; 218]	0.806
Protein (E%)	23 [22; 24]	20 [19; 22]	3 [1.4; 5]	**0.001**
Carbohydrate (E%)	42 [41; 44]	40 [38; 41]	2 [-0.6; 6]	0.111
Fat (E%)	31 [29; 33]	36 [34; 38]	-5 [-9; -2]	**0.002**
Dietary Fiber (E%)	3 [3; 4]	4 [3; 4]	-1 [-1; 1]	0.934

ESM Table 3. Macronutrient composition of breakfast and full-day diet in the early and late third trimester and random days in the third trimester. Data are presented as means with 95%CI. Women recorded their dietary intake during free-living conditions in the early and late third trimester and on random days between early and late third trimester and between late third trimester and delivery. The content of pre-meals is included in the full-day macronutrient composition but not in the breakfast. A mixed-effects model was used to analyse data followed by pairwise comparisons for differences between placebo and WP. E%, energy percentage.

**Outcome**	**WP, *n*=30**		**Placebo, *n*=25**		***p***
Initiation of insulin	7%	*n*=2	4%	*n*=1	1.00
Caesarean section	23%	*n*=7	16%	*n*=4	0.736
Instrumental	7%	*n*=2	8%	*n*=2	1.00
Hypertension	7%	*n*=2	24%	*n*=6	0.123
Preeclampsia	7%	*n*=2	20%	*n*=5	0.226
Premature	10%	*n*=3	4%	*n*=1	0.617
Hypoglycaemia	7%	*n*=2	8%	*n*=2	1.00
Shoulder dystocia	7%	*n*=2	4%	*n*=1	1.00
Hyperbilirubinemia	4%	*n*=1	4%	*n*=1	1.00

ESM Table 4. Obstetrical outcomes. Data is presented as proportions of women or newborns with occurrences in the WP group and the placebo groups. The absolute number of any outcome is given after the proportion as *n*. An unpaired t-test was used to compute p-values for differences between groups.

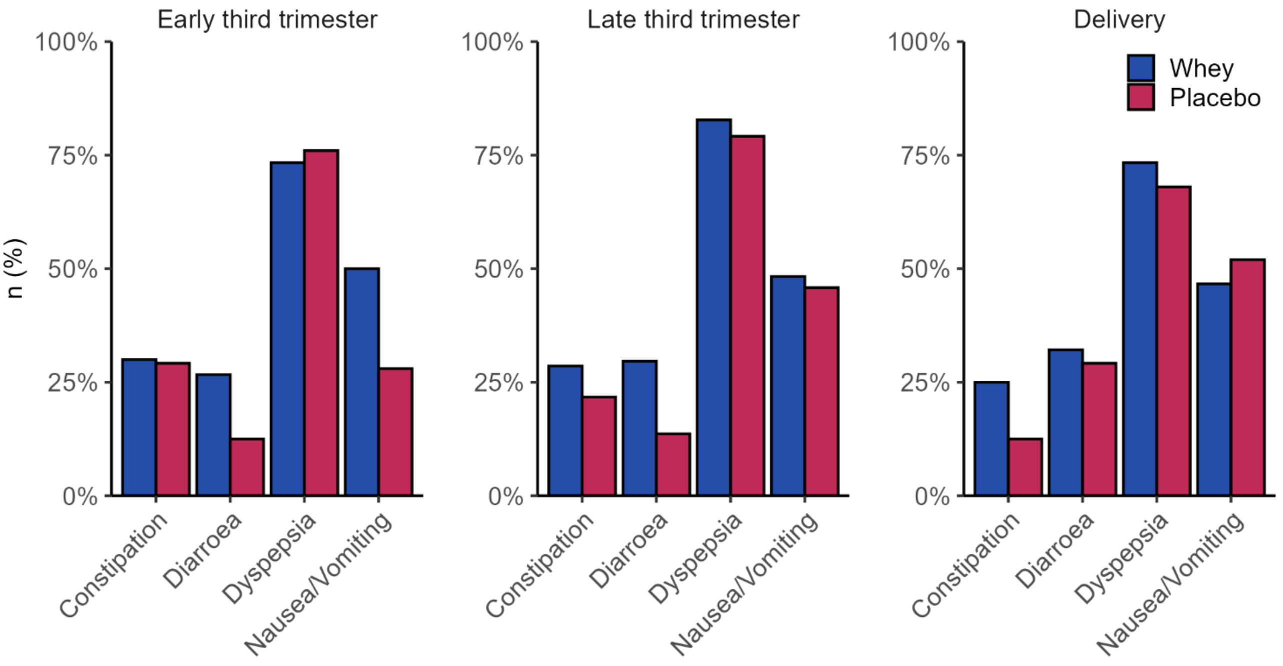

ESM Fig. 1. Gastrointestinal side effects. The first facet depicts the proportion of women (n (%)) who experienced gastrointestinal symptoms in the early third trimester at study entry. The second facet presents gastrointestinal symptoms during the late third trimester, and the last facet presents gastrointestinal symptoms up to delivery. Blue indicates pre-meal WP protein, and red indicates placebo. A mixed-effects model was used to analyse data followed by pairwise comparisons for differences between placebo and WP. There were no statistically significant differences.
